# Stereotypes of Women and Men Across Gender Subgroups

**DOI:** 10.3389/fpsyg.2022.881418

**Published:** 2022-04-28

**Authors:** Hege H. Bye, Vera V. Solianik, Martine Five, Mehri S. Agai

**Affiliations:** Department of Psychosocial Science, University of Bergen, Bergen, Norway

**Keywords:** gender stereotypes, warmth, stereotype content model, social role theory, intersectionality, competence

## Abstract

In this paper, we argue for the value of studying gender stereotypes at the subgroup level, combining insights from the stereotype content model, social role theory, and intersectional perspectives. Empirically, we investigate the stereotype content of gender subgroups in Norway, a cultural context for which a systematic description of stereotypes of gender subgroups is lacking. In a pilot study (*n* = 60), we established salient subgroups within the Norwegian context. Employing the stereotype content model, these groups were rated on warmth and competence in a main study (*n* = 191). Combining social role and intersectional perspectives, we compared stereotypes of women and men in the same social roles and social categories across subgroups. Comparisons between subgroups of women and men occupying the same social role indicated that at the subgroup level, women are often viewed as warmer than men, whereas the reverse appears to be a rare exception. Competence ratings, however, did not show this consistency. Our results at the subgroup level are consistent with research indicating that current gender stereotypes converge on constructs related to the competence dimension and remain divergent for constructs related to warmth.

## Introduction

Gender stereotypes are key to understanding a host of psychological phenomena, especially gender-based biases and discrimination. Within this thriving research field, three key issues are currently at the center of scientific inquiry: understanding how stereotypes are shaped by the intersection of gender with other social group memberships (i.e., intersectionality), understanding how gender stereotypes are rooted in specific social roles (e.g., occupational roles), and stability and change in gender stereotypes over time. Researchers interested in intersectionality or social roles often zoom in on the unique issues pertaining to specific gender subgroups (e.g., women and men of different ethnicities, Ghavami and Peplau, 2013; women and men in the same occupations, Gustafsson-Sendén et al., 2020). Researchers interested in change and stability over time tend to focus on women and men as broad, generic categories (Hentschel et al., 2019; Eagly et al., 2020; Bhatia and Bhatia, 2021). The merit of both approaches is unquestionable. However, the focus on either a specific intersection of gender with another social category or specific social roles, or on generic women and men, leaves that pattern of stereotype content across a wider range of gender subgroups unaddressed.

In this paper, we argue for the value of studying gender stereotypes at the subgroup level. Empirically, we investigate the stereotype content of gender subgroups in Norway, a cultural context for which a systematic description of stereotypes of gender subgroups is lacking. We draw on both the stereotype content model and social role theory, as well as intersectional perspectives, to address the stereotype content of gender subgroups. Specifically, we investigate the stereotype content of a range of respondent-generated subgroups of women and men in the Norwegian context, including groups defined by gender and social category membership (e.g., age and gender), gender and social role (e.g., female and male academics), and subgroups specific to either women (e.g., babes) or men (e.g., rockers). We particularly compare what we call *parallel gender subgroups*: women and men who either occupy the same social role or share a social category membership. We discuss what our results mean for the study of change and stability of gender stereotypes and cross-cultural approaches to gender stereotypes.

Across cultures, women have typically been stereotyped as more warm/communal (e.g., kind and nurturing), but less competent (e.g., intelligent and skillful) and agentic (e.g., ambitious, independent, and strong) than men (Williams and Best, 1982; Eagly et al., 2000; Wood and Eagly, 2012; Ellemers, 2018). While there is evidence that these gender stereotypes are resistant to change across time (Haines et al., 2016), there is also research showing that stereotypes of women and men increasingly overlap, especially on the competence dimension (Diekman and Eagly, 2000; Diekman et al., 2005; Gustafsson-Sendén et al., 2019). For example, based on analyses of public opinion polls in the US from 1946 to 2018, Eagly and colleagues demonstrated that women were increasingly seen as equally, or even more, competent than men. Women were, however, seen as consistently more communal and less agentic than men (Eagly et al., 2020).

Importantly, people hold stereotypes not only of the superordinate categories of “men” and “women,” but also about more specific gender subgroups (e.g., mothers and old men). A range of studies show that people can list and distinguish between the stereotypic traits of several female and male subgroups (Clifton et al., 1976; Eckes, 1994, 2002; Wade and Brewer, 2006; Athenstaedt et al., 2008). These subgroups often reflect the intersection of gender with other social roles such as occupation or parental status (Deux and LaFrance, 1998; Wood and Eagly, 2010) or the intersection of gender with other social categories such as race/ethnicity and sexual orientation (Ghavami and Peplau, 2013; Kang and Bodenhausen, 2015; Klysing et al., 2021).

Subgroups may be considered the “natural level of categorization for human targets because they provide more specific information than the superordinate category” (Wade and Brewer, 2006, p. 759, see also Pattyn et al., 2015). Haines et al. (2016) argued that when research participants rate the generic categories of women and men, we cannot know what specific images they have in mind when answering, and stereotypic elements may be stronger for some subgroups of women and men than for others in cases where gender intersects with other categories.

In their model of intersectional invisibility, Purdie-Vaughns and Eibach (2008) argue that ideologies of androcentrism, ethnocentrism, and heterosexism makes the prototypical human a man, the prototypical citizen (in a Western context) white, and heterosexuality prototypical of human sexuality. In combination, this makes prototypical women and men white and heterosexual. Moreover, the prototypical ethnic minority individual is a heterosexual man, and the prototypical homosexual person is a white man. This renders individuals with two subordinate social identities (e.g., ethnic minority women and ethnic minority gay men) to experience intersectional invisibility; they are not fully recognized as members of their groups.

Studies addressing stereotype content from an intersectional perspective largely support this model. For example, Ghavami and Peplau (2013) found that the stereotype content of “women” overlapped to a greater extent with “white women” than with “Middle Eastern American,” “Asian American,” “Latina,” or “Black” women. Similarly, they found that the overlap in stereotype content was largest between “men” and “white men” as compared to men of other ethnicities. In a study of the stereotype content of women, men, and women and men with different sexual orientations, Klysing et al. (2021, Study 1, p. 6) found that “general gender stereotypes only apply to heterosexual women and men.” Studies of stereotypes of typical women and men should therefore be complimented by studies that address gender stereotypes at the subgroup level. Research demonstrating changes in superordinate-level stereotypes of women and men also begs the question of whether and how these broad changes may be reflected at the level of subgroups.

There are several theoretical approaches to the study of stereotypes of gender subgroups. Following earlier research on subgroups of women and men (Eckes, 2002; Wade and Brewer, 2006), we take the Stereotype Content Model (SCM; Fiske et al., 2002; Fiske et al., 2007) as our starting point. In the SCM, warmth and competence^1^ are considered universal dimensions of social perception, along which stereotypes of social groups may be differentiated. Some groups are stereotyped as high or low on both dimensions, but many groups receive ambivalent stereotypes (i.e., high competence/low warmth or low competence/high warmth; SCM; Fiske et al., 2002; Fiske et al., 2007). The SCM is a general model of stereotype content and has been applied to a range of social groups; however, its emphasis on ambivalent stereotypes is particularly relevant to women and men as social categories. In fact, the SCM originated in research on ambivalent sexism (Glick and Fiske, 2011). According to ambivalent sexism theory, women fulfilling traditional roles (e.g., housewife and mother) are targets of benevolent sexism and paternalistic prejudice. Stereotyped as warm but incompetent, they are liked but disrespected. Women who challenge the status quo (e.g., feminists and career women) are targets of hostile sexism and envious prejudice. Stereotyped as competent but cold, they are respected for their competence but disliked (Glick and Fiske, 2001). Similarly, subgroups of men have also been found to be targets of both paternalistic prejudice (e.g., warm but incompetent “soft men”) and envious prejudice (e.g., competent and but cold male managers; Eckes, 2002).

In the SCM, stereotype content is theorized to stem from structural relationships between groups (Fiske et al., 2002). High-status groups are stereotyped as competent, low-status groups as incompetent. Well-intentioned, non-competitive groups are stereotyped as warm, groups that compete over scarce resources are viewed as cold. From a SCM perspective, women and men in the same social role or sharing another category membership may be stereotyped similarly or differently, depending on how the groups are assessed in terms of status and competition.

We also draw on social role theory (Eagly and Wood, 2012) which postulates that stereotypes of women and men are rooted in the division of labor between the sexes and distribution of women and men in different social roles. Unlike the SCM, a key prediction of social role theory is that role information overrides the effect of gender on stereotyping. Put differently, when women and men occupy the same social role, the effect of gender on inferences of stereotypical traits weakens or disappears (Eagly and Steffen, 1984; Bosak et al., 2012; Koenig and Eagly, 2014; Gustafsson-Sendén et al., 2020). For example, research showing that working mothers and working fathers (Cuddy et al., 2004) and male and female middle managers (Rosette and Tost, 2010) are stereotyped as equally warm and competent, supports this claim. However, there is also evidence that women and men in the same social role are stereotyped differently. Schneider and Bos (2014) found that stereotypes of male and female politicians differed; women were seen as lower in traits reflective of leadership, competence, and agency, and slightly higher in warmth. There are also instances in which the provision of role information has a counter-stereotypical effect, so that, for example, men described in a female-dominated role (i.e., a social role most commonly enacted by women) are seen as more communal than women in the same social role (Eagly and Steffen, 1984; Steinmetz et al., 2014).

What is clear from the above examples is that gender interacts with social roles with respect to stereotype content. However, it is more difficult to discern a systematic pattern in this process because each study compares stereotypes of women and men in a single or a limited number of social roles. There are studies of stereotypes of gender subgroups that include a whole range of male and/or female groups (Eckes, 1994, 2002; Wade and Brewer, 2006). However, these studies have not had a comparative focus addressing stereotypes of women and men in the same social roles. We combine these two approaches and look at stereotypes of subgroups of both women and men across several social roles (i.e., occupations, civil and parental status, and leisure roles).

Finally, we draw on intersectional perspectives. In their definition of intersectionality, Else-Quest and Hyde (2016, p. 156–157) emphasize that an intersectional perspective entails first a recognition that all people are characterized by multiple social identities (e.g., gender, race, sexual orientation, and age), that these categories are intertwined, and that the experience of each category is linked to the other categories. Second, embedded in these social categories is a dimension or aspect of inequality or power. Third, social categories are both properties of individuals (i.e., identity) and characterizations of social contexts (i.e., categories are constructed, and power inequalities are enforced by social structures, institutions, and interpersonal interactions). Stereotypes, as collective and shared cultural images of characteristics of social groups, form part of individuals’ social contexts and thus likely shape individuals’ experiences of what it means to be a member of a specific social group.

In theoretical scope, intersectional perspectives reach far beyond the domain of stereotypes. But, of relevance to the present study, intersectional perspectives also speak to how gender intersects with other social categories to form stereotypes (Crenshaw, 1989; Cole, 2009; Kang and Bodenhausen, 2015). When stereotypes have been studied from an intersectional perspective, researchers have studied mainly intersections of gender and race, as well as sexual orientation and age (Kang and Bodenhausen, 2015; Klysing et al., 2021). Unlike social roles, which are theorized to override the effect of gender on stereotype ascriptions, important social categorizations such as age groups and race/ethnicity combine with gender to produce stereotypes of subgroups of women and men that are more than—or rather qualitatively different from—the simple sum of each category’s constituent parts (Crenshaw, 1989; Cole, 2009; Ghavami and Peplau, 2013; Kang and Bodenhausen, 2015; Rosenthal and Lobel, 2016). A study of gender subgroups needs therefore include subgroups formed by both gender and social roles, and gender and social categories.

To summarize, our claim is not that stereotypes of gender subgroups have not been studied previously. Rather, the first contribution of this study is to combine the simultaneous study of *many* gender subgroups (building on the stereotype content model), with the study of parallel gender subgroups across social roles (building on social role theory) and across social categories (intersectionality). The second contribution is to provide empirical data from a cultural context—Norway—for which a systematic description of gender subgroup stereotypes is lacking.

Most studies on gender stereotypes have been conducted in the United States (but see Gustafsson-Sendén et al., 2019 and Klysing et al., 2021 for recent studies in the Swedish context) and expanding research to more cultural contexts had been identified as an important avenue for research (Sczesny et al., 2019). In their review of the literature, Athenstaedt et al. (2008) point to similarities in common gender subgroups and their associated stereotype content in Western societies. However, one would also expect some variations across cultures because gender stereotypes are shaped by both cultural values and the social roles enacted by women and men within a society (Wood and Eagly, 2012; Steinmetz et al., 2014; Cuddy et al., 2015). From both these perspectives, Norway provides an interesting context. With respect to cultural values, Hofstede’s dimension of masculinity-femininity appears particularly relevant. In highly feminine cultures, there is comparatively less social role differentiation between women and men, and sympathy and caring for others are viewed as desirable among both women and men. On this dimension, Norway has been ranked as one of the world’s most feminine cultures (Hofstede, 2011). This cultural value emphasis on femininity is consistent with Norway’s ranking on the global gender gap index (World Economic Forum, 2020). In 2020, Norway was ranked the second most gender equal country in the world, below Iceland ranked first. The other Nordic countries followed at rank 3 and 4 (Finland and Sweden) and 14 (Denmark). On some central indicators of women’s economic status and power, such as labor market participation (73% vs. 67.2% for men and women, respectively) and political representation (members of parliament: men 55%; women 45%), the figures are approaching an equal distribution (Statistics Norway, 2019, 2021).

Men’s roles are also becoming more equal to those of women, especially in the family domain. Although mothers take longer parental leaves than fathers, in 2017, 70% of fathers stayed at home during the weeks of parental leave reserved for them (10 weeks in 2017) or took an even greater share of the total parental leave period (Statistics Norway, 2017). Thus, the present study was conducted within a national and cultural context of high, but far from perfect, equality between women and men.

## Materials and Methods

To investigate stereotypes of subgroups of women and men, we first conducted a pilot study to compile a list of contemporary gender subgroups in Norwegian society. Next, in the main study, subgroups identified in the pilot study were rated on warmth and competence.

### Pilot Study

Participants (*N* = 60) were approached in public places in the city center of Bergen, Norway, and asked to take part in a short survey about which groups they experience that women and men in society are divided in to. Among the participants, 50.0% were women. A 48.3% were men (one participant did not answer the question about sex/gender^2^) and the mean age was 29.58 (*SD =* 13.75, range 18–79). The majority (90.0%) did not have an immigrant background. The remainder had either immigrated themselves (6.7%) or were Norwegian born to one or two immigrant parents (1.7%). One participant did not answer the background question.

The instruction to the participants was as: ‘There are many different “types” of women in today’s society. Please write down as many different types of women (e.g., mothers of small children, businesswomen) as you can think of.’ The rest of the page was left blank for the participant to write on. On a separate page, an identical instruction referring to types of men was presented. The order of presentation was counterbalanced so that half the participants listed women first, the other half listed men first.

On average, the participants listed 7.28 different types of women (*SD* = 4.07, range 0–21) and 7.42 different types of men (*SD* = 4.47, range 0–24). Three criteria were employed to determine which groups would be chosen as stimulus groups in the main study: (a) the group had to be mentioned by at least four participants, (b) we sought to have groups from different social roles and categories (e.g., groups defined by family and professional roles and sexual orientations), and (c) we wanted to ensure that we had parallel male and female subgroups (e.g., male politicians and female politicians, single mothers, and single fathers) to facilitate comparisons of men and women occupying the same social role and category. Our choice that a groups should be mentioned by four participants was informed by prior published work (e.g., Lee and Fiske, 2006; Durante et al., 2013) but was also pragmatic in the sense that a stricter criterion would limit the number of groups included and a more lenient one would involve too many groups to be rated.

Based on these criteria, 19 male subgroups (business men, fathers of small children, police- and firemen, bachelors, rich men, work men, soft men, male students, outdoorsy men, single fathers, handy men, single men, male leaders, old men, gay men, male academics, sporty men, male politicians, and rockers) and 22 subgroups of women (single mothers, female politicians, career women, mothers of small children, nurses, feminists, female students, outdoorsy women, teachers (the female version of the word in Norwegian was used), lesbians, old ladies, bloggers, single women, immigrant women, macho women, babes, female artists, exercise women, female academics, fashion women, housewives, and female leaders) were selected.

Choosing groups in part based on their salience in the pilot sample is not without risks. As Fiske et al. (2002) described, both groups eliciting antipathy and in-groups may be less likely to appear. We recognize that our list of groups is limited in several ways: For example, the list does not contain a full range of sexual orientations (e.g., bisexual women or men are excluded, heterosexuality was not mentioned in the pilot and presumably taken for granted), and “immigrants” were used rather than specifying specific ethnicities or countries of origin. We are not suggesting that our list is representative of the subgroups that women and men may belong to. However, the groups selected are salient in the Norwegian context, and many of the included gender subgroups have been identified in previous research in other national contexts (Athenstaedt et al., 2008).

### Main Study

#### Participants and Procedure

Similar to the pilot study, participants^3^ (*N* = 191) were approached in public places. We collected data in 2014 and 2015, in a town in Northern Norway, in a municipality outside Bergen, and in the Oslo-region, in addition to Bergen city center. Participants were asked to take part in a short survey about how different types of men and women are regarded in Norwegian society. Participants were explicitly instructed to indicate how they believed the groups were viewed by most people, and not to give their personal opinions.

Among the participants, 43.5% were men, 56.0% were women (one participant did not answer the question about sex/gender), and the mean age was 36.84 years (*SD =* 16.32, range 17–82). The majority (81.1%) did not have an immigrant background. The remainder had either immigrated themselves (9.4%) or were Norwegian born to one or two immigrant parents (5.8%). Four participants indicated “other background,” three indicated that they did not want to respond to this question, and one did not answer the background question.

#### Measures

Based on the pilot study, the participants rated 42 subgroups of women and men on warmth and competence.^4^ This part of the questionnaire consisted of four parts (lists of subgroups of women and men to be rated on warmth and competence), whose order was randomized. Similar to the procedure in Lee and Fiske (2006), one question measured perceived warmth and competence, respectively. For each list of subgroups, the respondents were asked to “think about how the different groups of women [men] listed below are perceived by people in Norway in general. To what extent do most people view each of the groups as (a) warm (friendly, good natured, and sincere) and (b) competent (confident, capable, and skillful)?” The items were responded to on a scale from 1 (Not at all) to 5 (To a very large extent). Single-item measures of warmth and competence were chosen to allow for a design in which all participants rated all groups. To off-set some of the limitations of using single-item measures, we included additional characteristics in each item (as described above) to convey to the participants the breadth and intended meaning of the “warmth” and “competence” constructs.

#### Preliminary Analyses

As a preliminary analysis, we explored whether stereotype ratings differed systematically between women and men. We conducted a series of independent samples (*t*) tests (two tailed) and corrected the *p*-values to control for the familywise error rate with the Holm-Bonferroni correction (Holm, 1979; Gaetano, 2018). Across the in total 84 ratings of subgroup warmth and competence, the ratings made by women and men did not differ significantly (*p* > 0.05) in 82 instances (97.6%), suggesting that perceptions of societal subgroup stereotypes are largely similar among women and men. The exception to this pattern was that men rated feminists as significantly less warm and less competent than women did. Given the similarity in women and men’s ratings of the subgroups, our main analyses are not stratified by participant sex/gender.

## Results

### Stereotypes of Subgroups of Women and Men

**Figure 1:**
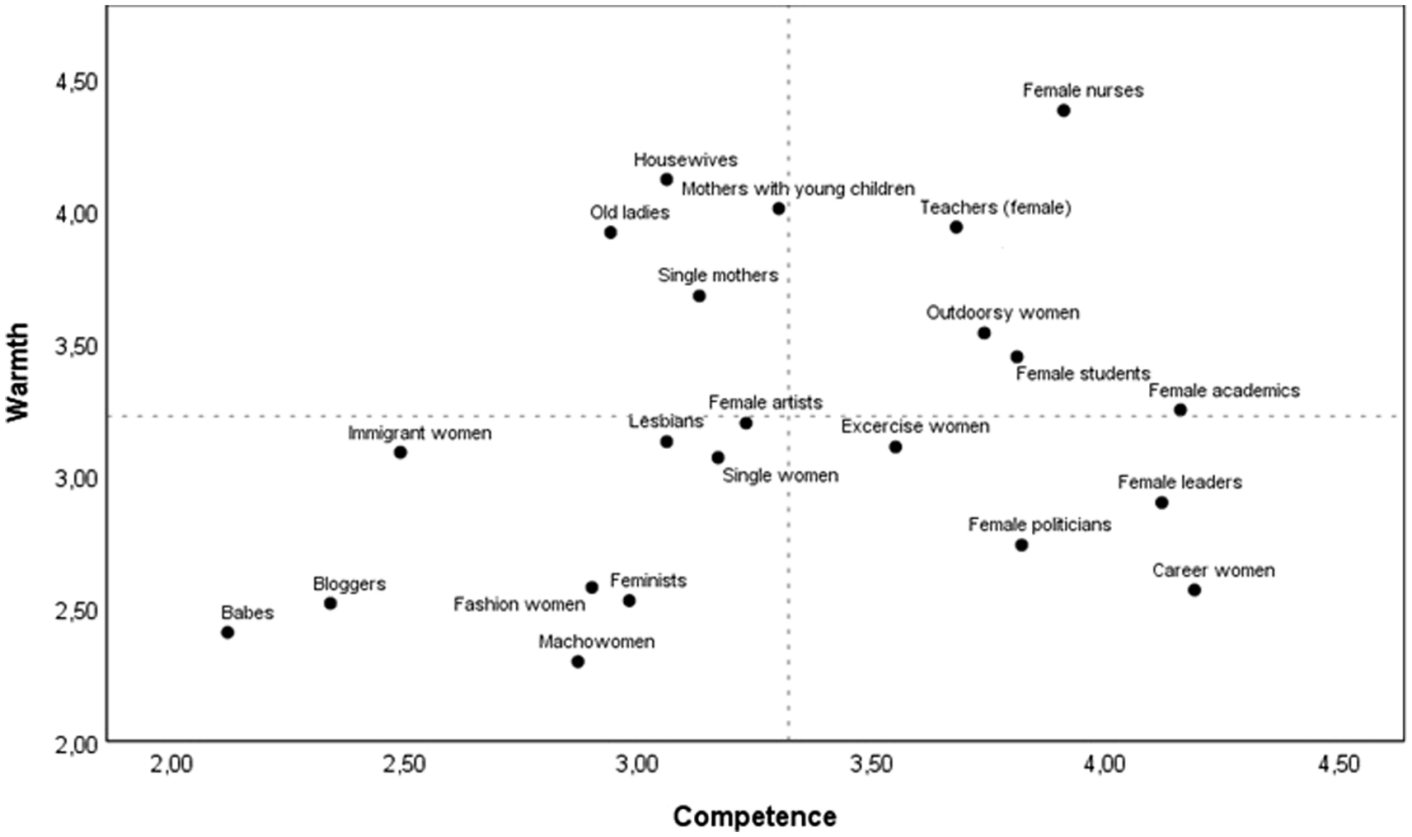
Means of warmth and competence for subgroups of women. Dotted lines indicate grand means across subgroups. Please note that the axes in the figure have been truncated.

First, we provide an overview of the stereotype content of subgroups of women and men across all the subgroups included in the study. The stereotype content of subgroups of women is presented in Table 1 and Figure 1. Table 1 presents the ratings of warmth and competence for each subgroup of women, and paired samples *t*-tests (two tailed) comparing each group’s warmth and competence corrected for multiple testing by the Holm-Bonferroni correction (Holm, 1979; Gaetano, 2018). With only three exceptions (single women, lesbians, and female artists), all subgroups of women were ambivalently stereotyped. Figure 1 shows that housewives, old ladies, mothers with young children, and single mothers are similarly stereotyped as warm, but not so competent. Female politicians, female leaders, and career women clustered together as competent, but not warm. Babes and bloggers stand out as subgroups stereotyped as cold and incompetent, joined by fashion women, feminists, and macho women in the cold, but not competent quadrant of the SCM space. Immigrant women were rated as average in warmth, but as incompetent. Stereotypes of lesbians, female artists, single women, and exercise women were located in the middle of the SCM space, indicating more moderate perceptions of both warmth and competence. In the warm and competent quadrant of the SCM space, where previous research indicates that “women” as a generic category are located in the Norwegian context (Bye et al., 2014), we find groups based on two very common professional roles for women, nurses, and teachers, but also female students and outdoorsy women.

**Table 1:** Warmth and competence means, standard deviations, and paired samples *t*-tests for subgroups of women.

Subgroup	Warmth		Competence		*df*	*t*	*p*	Cohen’s *d_z_*
	*M*	*SD*	*M*	SD				
Career women	2.56	0.86	4.18	0.76	185	−20.062	0.000	−1.47
Female politicians	2.74	0.89	3.82	0.76	185	−14.628	0.000	−1.07
Housewives	4.12	0.70	3.05	0.97	185	13.317	0.000	0.98
Female academics	3.25	0.74	4.16	0.68	185	−13.025	0.000	−0.96
Old ladies	3.93	0.91	2.93	0.94	185	12.231	0.000	0.90
Female leaders	2.90	0.89	4.11	0.76	114	−13.440	0.000	−1.25
Mothers with small children	4.02	0.72	3.28	0.77	185	11.128	0.000	0.82
Immigrant women	3.09	0.85	2.47	0.87	185	9.561	0.000	0.70
Macho women	2.31	0.81	2.87	0.89	185	−8.675	0.000	−0.64
Single mothers	3.69	0.83	3.14	0.90	184	8.203	0.000	0.60
Female nurses	4.38	0.67	3.91	0.82	185	7.807	0.000	0.57
Exercise women	3.12	0.72	3.55	0.77	184	−6.930	0.000	−0.51
Female students	3.45	0.66	3.82	0.73	185	−6.053	0.000	−0.51
Feminists	2.53	0.93	2.98	0.91	185	−6.053	0.000	−0.44
Babes	2.42	0.90	2.12	0.91	182	4.651	0.000	0.34
Fashion women	2.58	0.84	2.90	0.91	184	−4.267	0.000	−0.31
Female teachers	3.95	0.72	3.68	0.75	185	4.085	0.000	0.30
Bloggers	2.51	0.82	2.33	0.94	183	2.618	0.048	0.19
Outdoorsy women	3.54	0.80	3.74	0.77	185	−2.561	0.048	−0.19
Single women	3.08	0.85	3.17	0.76	184	−1.309	0.576	−0.10
Lesbians	3.14	0.93	3.06	0.87	184	1.222	0.576	0.09
Female artists	3.22	0.74	3.23	0.92	185	−0.160	0.873	−0.01

*Values of p are corrected using the Holm-Bonferroni correction for multiple testing*.

**Figure 2:**
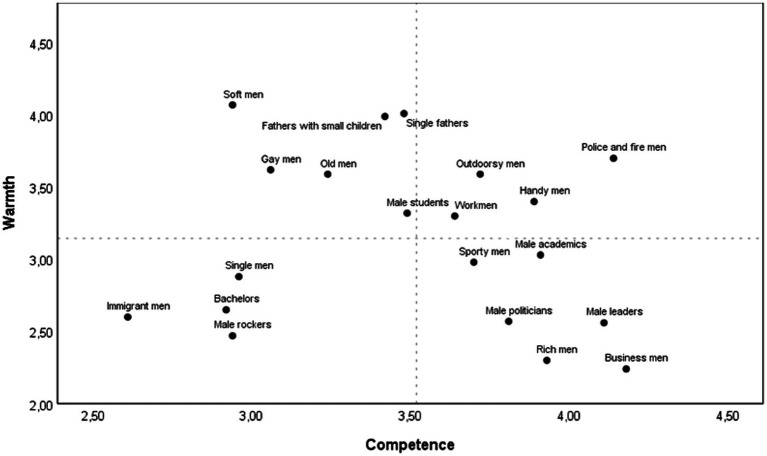
Means of warmth and competence for subgroups of men. Dotted lines indicate grand means across subgroups. Please note that the axes in the figure have been truncated.

Table 2 presents the ratings of warmth and competence for each subgroup of men, and paired samples *t*-tests (two tailed) comparing each group’s warmth and competence corrected for multiple testing by the Holm-Bonferroni correction (Holm, 1979; Gaetano, 2018). Stereotypes of subgroups of men along the warmth and competence axes are plotted in Figure 2. Like the results for the subgroups of women, male politicians, leaders, businessmen, and rich men were stereotyped as competent but cold. Men described as single, bachelors, rockers, or immigrants were viewed as cold, and less competent, although competence scores were significantly higher than warmth ratings for rockers and bachelors. Handy men, work men, sporty men, male students, and male academics were stereotyped as competent and moderately warm, similar to the superordinate category of men in previous research in Norway (Bye et al., 2014). Both fathers of small children and single fathers, along with soft men, were perceived as particularly warm, but less competent. These groups were in the warm but incompetent quadrant of the SCM space, along with gay men and old men.

**Table 2:** Warmth and competence means, standard deviations, and paired samples *t*-tests for subgroups of men.

Subgroup	Warmth		Competence		*df*	*t*	*p*	Cohen’s *d_z_*
	*M*	*SD*	*M*	SD				
Businessmen	2.23	0.74	4.17	0.73	−25.421	184	0.000	−1.87
Fathers with small children	4.00	0.75	3.41	0.69	9.668	183	0.000	0.71
Police/firemen	3.70	0.88	4.13	0.75	−6.442	184	0.000	−0.47
Bachelors	2.65	0.74	2.90	0.77	−3.843	183	0.001	−0.28
Rich men	2.28	0.83	3.92	0.87	−19.160	184	0.000	−1.41
Work men	3.30	0.80	3.63	0.77	−5.014	183	0.000	−0.37
Soft men	4.08	0.79	2.94	0.84	13.630	184	0.000	1.00
Male students	3.32	0.70	3.49	0.78	−2.801	184	0.023	−0.21
Outdoorsy men	3.60	0.85	3.71	0.80	−1.608	183	0.329	−0.12
Single fathers	4.01	0.79	3.46	0.88	7.683	184	0.000	0.57
Handy men	3.40	0.76	3.88	0.74	−7.522	184	0.000	−0.55
Single men	2.88	0.83	2.93	0.82	−0.741	184	0.919	−0.06
Male leaders	2.55	0.90	4.09	0.82	−17.443	183	0.000	−1.29
Old men	3.60	0.85	3.23	0.97	4.463	183	0.000	0.33
Gay men	3.63	0.93	3.07	0.84	8.228	182	0.000	0.61
Male academics	3.03	0.75	3.92	0.83	−11.759	184	0.000	−0.87
Sporty men	2.98	0.78	3.70	0.81	−10.413	184	0.000	−0.77
Male politicians	2.57	0.90	3.80	0.84	−14.352	184	0.000	−1.06
Rockers	2.46	0.89	2.93	0.93	−5.448	183	0.000	−0.40
Immigrant men	2.60	0.94	2.60	0.86	0.000	114	1.000	0.00

*Values of p are corrected using the Holm-Bonferroni correction for multiple testing*.

Together, Figures 1 and 2 provide an overview of stereotypes of subgroups of women and men in Norway.

### Women and Men in the Same Social Roles and Categories Across Subgroups

Next, we compared the stereotypes of women and men in the same social roles and categories across several subgroups. Means and standard deviations are presented in Table 3. Paired samples *t*-tests (two tailed), again corrected for multiple testing by the Holm-Bonferroni correction (Holm, 1979; Gaetano, 2018), indicated that warmth ratings of subgroups of women were significantly higher than those of subgroups of men for five out of 12 comparisons (career women, old ladies, immigrant women, female academics, and single women were all rated as relatively warmer than their parallel male subgroups). In six instances, warmth ratings of parallel subgroups of women and men did not differ significantly (mothers and fathers with small children, female and male leaders, female and male students, female and male politicians, exercise/sporty women and men, and outdoorsy women and men). The only comparison in which the warmth rating of a subgroup of men was significantly higher than that of a subgroup of women was in the case of single parents: single fathers were rated as warmer than single mothers.

**Table 3:** Means, standard deviations, and comparisons of warmth and competence ratings for parallel subgroups of women and men.

Groups	Warmth					Competence				
	Subgroup of women *M (SD)*		Subgroup of men *M (SD)*	*n*	Cohen’s *d_z_*	Subgroup of women *M (SD)*		Subgroup of men *M (SD)*	*n*	Cohen’s *d_z_*
Single mothers vs. fathers	3.69 (0.82)	<	4.01 (0.79)	185	−0.37	3.13 (0.90)	<	3.48 (0.89)	188	−0.33
Mothers vs. fathers with small children	4.02 (0.72)	=	3.99 (0.75)	186	0.03	3.30 (0.77)	=	3.42 (0.69)	187	−0.15
Single women vs. men	3.06 (0.85)	>	2.88 (0.82)	186	0.20	3.17 (0.76)	>	2.96 (0.84)	187	0.24
Female vs. male academics	3.24 (0.73)	>	3.03 (0.75)	186	0.27	4.16 (0.68)	>	3.91 (0.83)	188	0.31
Female vs. male students	3.44 (0.65)	=	3.31 (0.70)	186	0.17	3.81 (0.73)	>	3.49 (0.78)	188	0.39
Career women vs. businessmen	2.55 (0.85)	>	2.24 (0.74)	186	0.38	4.19 (0.76)	=	4.18 (0.73)	188	0.01
Female vs. male leaders	2.90 (0.90)	=	2.69 (0.97)	116	0.19	4.12 (0.76)	=	4.05 (0.81)	117	0.09
Female vs. male politicians	2.73 (0.89)	=	2.57 (0.89)	186	0.16	3.82 (0.76)	=	3.81 (0.84)	188	0.01
Old ladies vs. old men	3.92 (0.91)	>	3.59 (0.85)	186	0.32	2.94 (0.94)	<	3.25 (0.98)	187	−0.34
Immigrant women vs. men	3.03 (0.87)	>	2.58 (0.91)	116	0.52	2.47 (0.89)	**=**	2.61 (0.86)	118	−0.18
Exercise women vs. sporty men	3.10 (0.72)	=	2.98 (0.79)	186	0.15	3.55 (0.78)	**=**	3.71 (0.81)	187	−0.19
Outdoorsy women vs. men	3.53 (0.79)	=	3.59 (0.85)	186	−0.07	3.73 (0.77)	**=**	3.73 (0.81)	187	0.01

**Figure 3:**
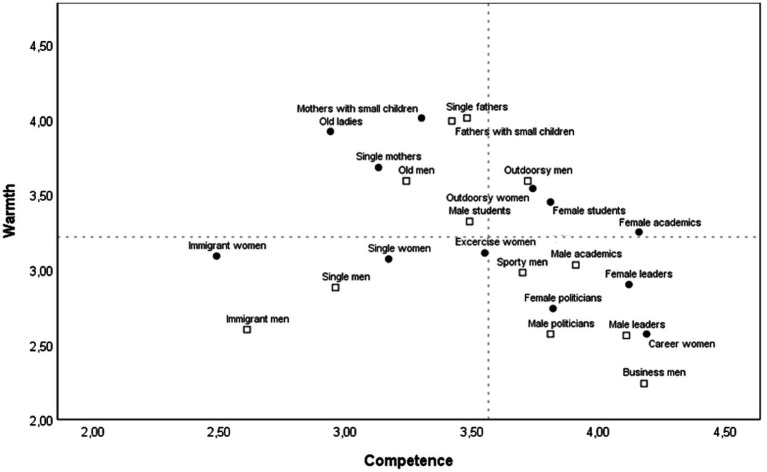
Means of warmth and competence for parallel subgroups of women and men. Squares represent subgroups of men; dots represent subgroups of women. Dotted lines indicate grand means across subgroups. Please note that the axes in the figure have been truncated.

Subgroups of men were rated as more competent than the parallel subgroup of women in two out of 12 comparisons (single fathers and old men were rated as more competent than their parallel female subgroups). In seven comparisons, there was not a significant difference in competence ratings of parallel subgroups (career women and businessmen, female and male leaders, female and male politicians, outdoorsy women and men, exercise women and sporty men, immigrant women and men, and mothers and fathers with small children). Finally, in three comparisons, subgroups of women were rated as higher in competence (single women, female academics and female students versus single men, male academics, and male students, respectively). The position of the parallel subgroups of women and men is presented in Figure 3.

## Discussion

Our results provide an overview of stereotypes of gender subgroups in a Norwegian context, answering calls for research beyond the US context (Sczesny et al., 2019). Despite the country’s ranking as one of the world’s most gender equal nations (World Economic Forum, 2020), our results to a large extent resonate with extant research on gender stereotypes (Ellemers, 2018), ambivalent sexism (Glick and Fiske, 2001, 2011), and social role theory (Eagly and Wood, 2012; Koenig and Eagly, 2014). Women and men in care-giving roles traditionally reserved for women are depicted as stereotypically warm and low in competence. Both women and men in traditional male roles (leaders, politicians, and businesspeople) are stereotypically competent and low in warmth. Our results regarding sexual minorities align with other research in finding stereotypes of gay men to indicate high warmth and moderate competence (Mize and Manago, 2018; Klysing et al., 2021). With respect to stereotypes of lesbians, we found moderate ascriptions of both competence and warmth, in line with Klysing et al., 2021 (Study 2). Others have indicated stereotypes of lesbians to ambivalent, with higher scores on competence/agency than warmth (Klysing et al., 2021, Study 1; Mize and Manago, 2018). This may suggest that there is variation in the perceptions of lesbians across national settings, but differences across samples could also reflect methodological issues, such as which other groups participants have rated as these may serve as anchors for the ratings of lesbians.

Moreover, we found that subgroups of women defined by their appearance (“babes”), who make a living in part from their appearance (“bloggers”) or show a marked interest in their appearance (“fashion women”), are likely targets of so-called contemptuous prejudice (Fiske et al., 2002) in Norwegian society. Here, our results correspond with research demonstrating that women facing sexualized or appearance-based objectification are viewed as lower in traits reflective of agency/competence and warmth than non-objectified women (Morris et al., 2018). More specific to the Norwegian context, perhaps, our results show that being outdoorsy appears to be culturally normative for both women and men. This peculiarity noted, our results point to cross-cultural similarity of gender stereotypes at the subgroup level, both in terms of which subgroups were mentioned and to some extent in their associated contents. A systematic cross-cultural study of gender stereotypes at the subgroup level would however be necessary to ascertain this empirically.

We also compared the warmth and competence ratings of women and men occupying the same social roles or sharing a social group membership. The results of these comparisons tell us four important things. First, also at the subgroup level, women are viewed as either warmer, or at least equally warm, compared to men. We documented only one exception to this general pattern, in the case of single fathers being viewed as warmer than single mothers. Thus, although our study was conducted in a different national context than that of Eagly et al. (2020), our results at the subgroup level are generally consistent with their results concerning the continued stereotyping of women in general as warmer than men. Gustafsson-Sendén et al., (2019) raised the question as to whether communal traits are harder to gain (stereotypically) for those not belonging to the category “woman.” They found that increasing men’s participation in communal roles (i.e., taking parental leave) did not lead to men in general being perceived as more communal. Our results suggest that it is not the case that *subgroups* of men cannot be perceived as warm: we found that men are viewed as warm (and at least as warm as women) when they occupy the role of father or enter professions that involve the protection of others (police and firemen). Similarly, other researchers have also found that stereotypes of fathers are dynamic and increasingly include “maternal” (warm) traits (Banchefsky and Park, 2016). Rather, our results suggest that warmth appears to be harder for women to lose. When women enter traditionally masculine roles, they are seen as colder than women in general, but not as cold as men in the same role. We find this pattern for career women, female academics, and (albeit not statistically significantly) female leaders and politicians. Paralleling our findings in their study of perceptions of parents, Banchefsky and Park (2016) found that although women and men’s roles as parents were seen as changing, women’s perceived maternal (warm) traits were not perceived to change.

That said, it is not that case that subgroups of women cannot be perceived as cold. The clearest example of this in our data is the subgroup “macho women,” but also other groups of women were rated as relatively cold (e.g., feminist and career women). To the extent that warmth stereotypes can be seen as parallels to femininity or reflective of womanhood, warmth is not indiscriminately ascribed to all types of women in an essentialist way. Relating our findings to the discussion about manhood and womanhood as achieved statuses (Chrisler, 2013; Vandello and Bosson, 2013), an interesting path for future research would be to investigate the perceived gender status (womanhood/manhood) of gender subgroups.

Second, our results indicate that competence ratings do not show the same consistency as ratings of warmth. Depending on the subgroups in question, the subgroups of women were rated as lower, equal, or higher in competence than the parallel subgroups of men. In roles associated with academia, politics, leadership, and business, women were rated as high or higher in competence than men. Men were rated as more competent than their female counterparts in the case of old men and single fathers. Again, this is consistent with the results of Eagly et al. (2020) with respect to women in general being perceived as equally (or more) competent as men. Thus, for both warmth and competence, our results at the subgroup level are broadly consistent with research indicating that current stereotypes of women and men converge on constructs related to the competence dimension and remain divergent for constructs related to warmth (Diekman and Eagly, 2000; Gustafsson-Sendén et al., 2019; Eagly et al., 2020). Martin and Slepian (2021, p. 1149) argue that “conceptions of competence vary according to the demands of the environment and thus easily shift depending on the goals that become valued.” Competence, they argue is a fundamental dimension of social perception “only to the extent that it is conflated with masculinity, dominance, and the agentic goals that have previously been needed and valued in the course of human evolution.” This could be an explanation of the seemingly higher degree of flexibility in competence perceptions. Future research should explore how intersecting gender with social roles and social categories impact on the ascription of traits and characteristics in the broader domain of agency/masculinity.

Third, our results show that when taking a broader perspective, women and men in the same social role were in the near vicinity of each other in the SCM space (see Figure 3). This is also true of the cases in which women and men in the same social role were rated as differing significantly in warmth or competence. Irrespective of their gender, businesspeople are broadly stereotyped as competent, but cold; parents are warm and academics competent. This attests both to the pervasiveness of the impact of role information on gender stereotypes (Eagly and Steffen, 1984; Eagly and Wood, 2012) and to the usefulness of studying stereotypes of gender subgroups within the general framework of the Stereotype Content Model (Fiske et al., 2002; Fiske et al., 2007). It also fits well with the emphasis in intersectional perspectives on the dynamic and fluid nature of social categorizations or positions (Else-Quest and Hyde, 2016). The characteristics associated with women and men are closely related to, and vary across, other category memberships (e.g., age and sexual orientation) and social roles (e.g., professional roles and parental status).

Fourth, for the subgroups created by the crossing of two social group memberships (i.e., gender and immigration status and gender and age), there is a tendency of larger effect sizes when comparing stereotypes of subgroups of women and men, than what we generally observed for subgroups created by the intersection of gender with a social role. Our study contained too few social category-by-gender subgroups to establish whether this is a consistent pattern, but it may be: Whereas social roles are expectations to incumbents of a social position in specific settings (e.g., at work), social group memberships based on demographic characteristics (e.g., age) have trans-situational implications (Koenig and Eagly, 2014, p. 372). How gender stereotypes are influenced may vary depending on whether gender is combined with a social group membership or with a social role. This could be investigated in future research by explicitly comparing subgroups created by combining gender with roles (e.g., gender and leadership), other social group memberships (e.g., gender and age), and both a role and a group membership (e.g., gender, age, and leadership). Whether gender, role, or other social category memberships more strongly impact stereotype ratings may also be impacted by individual differences in gender essentialism (Lee et al., 2020) and could be investigated in future work.

A limitation of the present study is the use of convenience rather than random samples. This is partly mitigated by the fact that we collected data from several geographic locations and from the public rather than a student sample. Moreover, our preliminary analyses demonstrated similarities in the perceptions of society’s view of subgroups of men and women reported by women and men (as participants). This points to a lack of impact of demographic variables on ratings of gender stereotypes as seen from the perspective of society. In line with this assertion, in a recent review of research on the Stereotype Content Model, Fiske (2018, p. 69) writes:

*Because respondents report society’s views, this minimizes social desirability concerns, and it means that samples need not be representative, because everyone knows the society’s stereotypes of common groups (compare the representative sample in* Cuddy et al., 2007*, with the convenience samples in* Fiske et al., 2002*). Individual differences and in-group favoritism are rare.*

Another limitation is our focus on competence rather than the broader construct of agency (encompassing both assertiveness and competence; Abele et al., 2016). We have also focused exclusively on positive traits (warmth and competence) and did not include negative characteristics previously included in research on gender stereotypes (see, e.g., Diekman and Eagly, 2000). Considering the number of groups our participants had to rate, we were limited in the number of measures we could include. However, we recognize that including a broader set of measures would have allowed for a more fine-grained analysis.

We also opted for a design in which all participants rated all subgroups, with the advantage of increased statistical power compared to a between-groups design (i.e., where smaller subsets of participants rate a random subset of groups). This was the background for the decision to measure warmth and competence with single items, following Lee and Fiske (2006). We recognize that our measures may not cover the full breadth of the warmth and competence constructs, even though the inclusion of “friendly, good natured, and sincere” in parentheses following “warm” and “confident, capable, and skillful” following “competent” may have given out participants an acceptable understanding of warmth and competence as global constructs. An alternative approach would have been to employ a three-item or four-item measure of warmth and competence (as commonly seen in between-groups designs). With 42 groups, however, this would lead participants to make 252–336 ratings. Given the time required and repetitive nature of the task, we suspect that this would have caused participant fatigue and hurt the validity of the ratings.

There is also the issue of statistical power, given the moderate size of our sample. G*Power version 3.1.9.4 (Faul et al., 2007) was used to conduct a sensitivity analysis to investigate the effect sizes that could be detected in our data. The smallest *n* in the comparisons between parallel subgroups was 116. With that sample size, we could detect mean differences with an effect of *d_z_* = 0.26 at power = 0.80 (*α* = 0.05, two tailed). For most of our statistical tests, the sample size is ≥185. With a sample of 185, we could detect mean differences with an effect of *d_z_* = 0.21 at power = 0.80 (*α* = 0.05, two tailed).

Finally, our study addresses perceptions, and not behaviors directed toward, gender subgroups. The Behavior from Intergroup Affect and Stereotypes Map (Cuddy et al., 2007) describe how warmth and competence perceptions trigger different types of emotional prejudices and intergroup behaviors along axes of facilitation and harm. This, combined with insights into the prescriptive nature of gender stereotypes (Ellemers, 2018), may be used as a framework for comparing the facets of gender-based prejudice and discrimination women and men belonging to different social groups and enacting different roles may experience. Also studies in other disciplines point to the value of stereotype content for predicting social behaviors toward social groups more generally (e.g., Jenkins et al., 2018).

These limitations noted, we believe the present study has several contributions. We have provided a rich description of stereotypes of gender subgroups in Norway, adding to our knowledge about the Scandinavian context (Gustafsson-Sendén et al., 2019; Klysing et al., 2021) and answering calls for research in a broader range of cultural contexts (Sczesny et al., 2019). We have shown that stereotypes at the subgroup level are consistent with research indicating that current gender stereotypes converge on constructs related to the competence dimension and remain divergent for constructs related to warmth (Eagly et al., 2020), contributing to the understanding of stereotype change. Finally, our study shows the relevance of studying multiple gender subgroups simultaneously and combining the stereotype content model, social role theory, and intersectional perspectives in the study of gender stereotypes.

## Data Availability Statement

The raw data supporting the conclusions of this article will be made available by the authors, without undue reservation.

## Ethics Statement

Ethical review and approval was not required for the study on human participants in accordance with the local legislation and institutional requirements. Written informed consent for participation was not required for this study in accordance with the national legislation and the institutional requirements.

## Author Contributions

HB, VS, MF, and MA contributed to the conception and design of the study. VS, MF, and MA collected the data. HB performed the statistical analysis and wrote the first draft of the manuscript. All authors contributed to the article and approved the submitted version.

## Funding

The open access publication of this paper is funded by the University of Bergen.

## Conflict of Interest

The authors declare that the research was conducted in the absence of any commercial or financial relationships that could be construed as a potential conflict of interest.

## Publisher’s Note

All claims expressed in this article are solely those of the authors and do not necessarily represent those of their affiliated organizations, or those of the publisher, the editors and the reviewers. Any product that may be evaluated in this article, or claim that may be made by its manufacturer, is not guaranteed or endorsed by the publisher.

## Footnotes

^1^It is important to note that within the SCM framework, the labels warmth and competence are used, rather than the terms communion and agency commonly employed in research on gender stereotypes (Ellemers, 2018). Warmth, as operationalized within the SCM, is largely synonymous with communion, including both traits indicative of warmth/sociability and morality (e.g., sincere). The competence dimension may be viewed as a facet of agency, which also encompasses traits related more to assertiveness (Abele et al., 2016; Eagly et al., 2020).

^2^In Norwegian, the participants were asked to indicate “Kjønn.” This term is used both to refer to “sex” and “gender,” and does not distinguish between biological, social, cultural or identity aspects of sex and gender. The response options were “Man” and “Woman,” and we regret not including more response options to this question.

^3^For the main study, we collected data from two samples. The first sample (*n* = 70) was on average quite young and had a majority of participants who identified as women and we wanted to obtain a more age and gender-balanced total sample of respondents, which led to the expansion of data collection with a second sample (*n* = 121). Financial and pragmatic issues contributed to a gap of a few months between the collection of data from sample 1 and 2.

^4^The measures were identical in the two sub samples of the main sample, with two exceptions. Due to an unfortunate error, “female leaders” was not included in the main questionnaire in the first sub-sample and was added to the list of groups in the questionnaire in the second sub sample. While “immigrant men” were only mentioned twice in the pilot, and therefore not originally included, we took the opportunity to include this group in the second round of data collection to have a parallel subgroup to “immigrant women.”
